# Mesenchymal stem cells‐derived exosomes ameliorate intervertebral disc degeneration through inhibiting pyroptosis

**DOI:** 10.1111/jcmm.15784

**Published:** 2020-08-29

**Authors:** Jingwei Zhang, Jieyuan Zhang, Yunlong Zhang, Wenjun Liu, Weifeng Ni, Xiaoyan Huang, Junjie Yuan, Bizeng Zhao, Haijun Xiao, Feng Xue

**Affiliations:** ^1^ Department of Orthopedics Shanghai Fengxian District Central Hospital/Southern Medical University Affiliated Fengxian Hospital Shanghai China; ^2^ Department of Orthopedics Shanghai Sixth People’s Hospital Shanghai China

**Keywords:** exosomes, IVDD, miR‐410, MSCs, pyroptosis

## Abstract

Mesenchymal stem cell (MSCs)‐based therapies have shown a promised result for intervertebral disc degeneration (IVDD) treatment. However, its molecular mechanisms remain unclear. Exosomes involve cell‐cell communication via transference of its contents among different cells, and the present potential effect on cell death regulation. This study aimed to investigate the role of MSCs‐derived exosomes on IVDD formation. Here, we first found the NLRP3‐mediated nucleus pulposus cell (NP cell) pyroptosis was activated in the IVDD mice model and lipopolysaccharide (LPS)‐induced model. However, MSCs treatment could inhibit NP cell pyroptosis in vitro. We then isolated MSCs‐derived exosomes by differential centrifugation and identified the characteristics. Secondly, we investigated the function of MSCs‐derived exosomes on LPS‐induced NP cell pyroptosis. Finally, we presented evidence that MSCs‐derived exosomal miR‐410 was a crucial regulator of pyroptosis. Results showed that MSCs‐derived exosomes play an anti‐pyroptosis role by suppressing the NLRP3 pathway. Moreover, it suggested that this effect was attributed to miR‐410, which was derived from MSCs‐exosomes and could directly bind to NLRP3mRNA. In conclusion, for the first time, we demonstrated that MSCs‐exosome treatment may inhibit pyroptosis and could be a promising therapeutic strategy for IVDD.

## 1 INTRODUCTION

Intervertebral disc degeneration is an ageing‐related disease. Nowadays, it is characterized by the reduction of the extracellular matrix, inflammatory activation or cell senescence, thereby fail to support spine structure and function, leading to many spine‐related disorders such as low back pain and disc herniation.^1^ The healthy intervertebral disc (IVD) is a tissue that is composed of inner soft nucleus pulposus (NP) cells and surrounding fibrocartilaginous ring—annulus fibrosus (AF), as well as cartilage endplates. The NP cells could promote the production of extracellular matrix to maintain the IVD hydrated. Researchers believe NP cell dehydration triggers the loss of disc height, disc deformation, segmental instability and even pain generation.^2^ Further study has confirmed that inhibit NP cell apoptosis could slow the ECM degeneration to delay the progression of IVDD.^3^ Therefore, it is essential further to understand the cell death mechanisms of NP cells to investigate new therapeutic strategies for IVDD.

Pyroptosis is a form of programmed cell death, which is triggered by various inflammasomes such as NOD‐, LRR‐ and pyrin domain‐containing proteins (NLRP), AIM2‐like receptor proteins and tripartite motif‐containing proteins. Once these inflammasomes are stimulated, the downstream inflammatory caspase 1 or caspase 11 will be cleaved and further promoting cells to release pro‐inflammatory cytokines IL‐1β and IL‐18.^4^, ^5^ This process is involved in various diseases, such as alcoholic liver disease and cancer.^6^, ^7^ Previous studies have reported that many pro‐inflammatory molecules such as interleukin‐1β (IL‐1β) and interleukin‐17 take part in the progress of primary catabolic enzymes expression, and subsequently influence many kinds of substrates such as proteoglycans, collagens and gelatins, which is highly related to IVDD formation.^8^, ^9^, ^10^ To date, Hu et al first presented that NLRP3‐mediated NP cell pyroptosis take part in the IVDD process both in vivo and in vitro under the propionibacterium acnes stimulation.^11^ Moreover, Tang et al reported that NOD‐, LRR‐ and pyrin domain‐containing proteins3 (NLRP3) inflammasome could be activated in nucleus pulposus cells after H_2_O_2_ stimulation.^12^ Another group observed activation of NLRP3 in the patients, which indicated that the dysregulation of NLRP3 might play a vital role in the development of IVDD.^13^

The exosome is one type of extracellular vesicles with a diameter of 50‐100 nm, which is originated from multivesicular endosomes and released by almost all kinds of cells. It contents various molecules, including cytokines, proteins, lipids and non‐coding RNAs. It is noteworthy that exosomes take part in cell‐cell communication by transferring its contents among different cells. Therefore, exosomes have emerged as important mediators for multiple disease therapy.^14^, ^15^ Some groups have reported that exosomes could be used to restrain pyroptosis. For example, Singla et al found that doxorubicin exposure significantly stimulated the expression of NLRP3 to induce pyroptosis in H9c2 cells. With the treatment of exosomes derived from embryonic stem cells, pyroptosis could be inhibited.^16^ They further demonstrated that exosome treatment showed a similar effect in vivo, which is beneficial for ameliorating doxorubicin‐induced cardiomyopathy.^17^ Lu et al considered exosomes could be used as an alternative to stem cell therapy for IVDD. They found that stem cell–derived exosome treatment showed the same effect on NP cell proliferation with stem cell therapy.^18^ Further studies confirmed the MSCs‐derived exosomes have antioxidant and anti‐inflammatory effects, as well as alleviated NP cell apoptosis, which is a benefit for IVDD treatment.^19^ However, whether exosomes inhibit pyroptosis in IVDD remains unknown.

For this purpose, we developed an IVDD model using LPS‐treated NP cells in this study. Then, we evaluated the therapeutic ability of MSCs and related exosomes on NP cell pyroptosis. Our data indicated that miR‐410 was enriched in MSCs‐derived exosomes and could target NLRP3 to suppress NP cell pyroptosis to prevent IVDD.

## 2 MATERIALS AND METHODS

### 2.1 Animals

Wild‐type C57BL/6 mice were obtained from Charles River Laboratory Animal Technology Co. Ltd. The IVDD model was established by annulus fibrosus (AF) needle puncture, as described in the previous study.^20^ In brief, after general anaesthesia with ketamine (100 mg/kg), the coccygeal discs were punctured by a syringe needle for 10 seconds. The needle needs to pass through the AF into the NP tissue to depressurize the nucleus. Then, the discs were harvested after 6 weeks. The sham group was subjected to the same operation without puncture. All procedures were conformed to the Guide for the Care and Use of Laboratory Animal. There are two reasons why we choose this model. First, it has been proved that the structure of C57BL/6 mice's tail discs is similar to that in human lumbar discs.^21^, ^22^, ^23^ Second, the morphologic characteristics of our IVDD model are similar to many human age‐related IVDD features, especially the changes in NP cells.^24^, ^25^

### 2.2 H&E staining

IVD tissue was fixed in 4% paraformaldehyde for 48 hours, and then embedded by paraffin and cut into five‐micrometre sections. Then, the sections were incubated in haematoxylin solution for 15 minutes, followed by stained using eosin solution for 15 seconds. Finally, the slices were dehydrated, transparentized and sealed. The images were captured under an optical microscope (Olympus).

### 2.3 Immunohistochemistry staining

According to the manufacturer's instructions, the slices were blocked using 1% horse serum solution for 30 minutess at room temperature. The slices were then incubated with the primary antibody against NLRP3 (1:500, Abcam, ab214185) overnight at 4°C. After incubation with high sensitivity streptavidin‐HRP conjugate for 30 minutes, DAB/AEC chromogen solution was added. In the end, the slices were visualized with a microscope at the bright field. The IHC kit was purchased from CST.

### 2.4 Cell culture and treatment

NP cells were isolated and cultured according to the previous study.^18^ In brief, the NP tissues were isolated from lumbar IVD of the normal mice under a stereotaxic microscope. After washed with PBS, the NP tissues were cut into small pieces and digested with a 0.5% type II collagenase (Roche Diagnosis) for 2 hours. The cells were obtained and maintained in F12 basal medium containing 10% foetal bovine serum and 100 U/mL penicillin‐streptomycin in a humidified incubator containing 5% CO_2_.

Human MSCs (hMSCs), fibroblasts and 293 cells (American Type Culture Collection) were maintained in DMEM medium added by 10% foetal bovine serum and 100 U/mL penicillin‐streptomycin in a humidified incubator containing 5% CO_2_. Passages 3 and 5 of NP cells, MSCs, 293 cells and fibroblasts were used for further experiments.

### 2.5 LPS treatment

Lipopolysaccharide was purchased from MedChem Express. The NP cells were treated with LPS (5 mmol/L) for 24 hours according to the manufacture's protocol. Cells were then collected for further experiments.

### 2.6 Co‐culture of MSCs and NP cells

Before co‐culture, the NP cells were treated with LPS. Then, the cells were seeded on the lower chambers of transwell plates. The MSCs were plated on the upper chambers of the transwell plates. After 24 hours, the NP cells were harvested for further study. In one group, the GW4869 (20 µmol/L) was added into the culture media of MSCs and incubated for 48 hours before co‐culture. GW4869 was purchased from MedChem Express.

### 2.7 Enzyme‐linked immunosorbent assay (ELISA)

After different treatments, the NP cell culture supernatant was collected. The level of cytokines IL‐18 and IL‐1β was quantified using specific ELISA kits (Abcam) according to the manufacture's protocol. Three independent repeats were performed.

### 2.8 Western blot

Protein lysates of cell samples or NP tissues were prepared by RIPA lysis buffer plus 1% phenylmethanesulfonyl fluoride. The total protein concentrations were measured with a BCA protein assay kit (Santa Cruz). Proteins were then separated by 10% SDS‐PAGE and transferred onto polyvinylidene fluoride membranes (Millipore). The membranes were then blocked by 5% bovine serum albumin for 2 hours at room temperature, followed by incubation with primary antibodies at 4°C overnight. After that, the membranes were incubated with second antibodies (1:5000, Abcam, ab150077) at room temperature for 2 hours. Protein bands were then detected using an enhanced chemiluminescence kit (ThermoFisher) and quantified by Image J software. Antibodies against NLRP3 (1:2000, ab214185), IL‐18 (1:1000,ab71495), IL‐1β (1:2000, ab234437), Gasdermin D (GSDMD, 1:1000, ab219800), TSG101 (1:2000, ab125011), and GAPDH (1:5000, ab181602) were purchased from Abcam. Antibodies against CD9 (1:2000, #13403), CD81 (1:2000, #56039), CD63 (1:2000, #55051), GM130 (1:1000, #12480), Cleaved caspase‐1 (1:1000, #89332), cleaved GSDMD (1:1000, #50928) and Calnexin (1:1000, #2679) were purchased from CST.

### 2.9 Immunofluorescence

The cells were fixed using 4% paraformaldehyde for 20 minutes at room temperature, followed by permeation using the 0.1% Triton for 5 minutes. Cells were then blocked by 5% bovine serum albumin for 1 hour at room temperature, followed by incubation with the primary antibodies against NLRP3 (1:500, Proteintech, 19771‐1‐AP) or caspase‐1 (1:500, Proteintech, 22915‐1‐AP) at 4°C overnight. Finally, cells were incubated with second antibodies (1:500, Abcam, ab150077) for 1 hour and stained with 4′,6‐Diamidine‐2′‐phenylindole dihydrochloride (DAPI, Roche) for 5 minutes at room temperature. Images were captured by a fluorescence microscope.

### 2.10 Isolation of exosomes

The MSCs and fibroblasts were washed by PBS for several times and cultured in DMEM medium supplemented with 10% exosome‐free foetal bovine serum for 48 hours. The culture medium was collected and centrifuged at 300 × *g* for 15 minutes at 4°C, followed by centrifugation at 2500 × *g* for 30 minutes. The supernatant was then filtered and ultra‐centrifuged at 100 000 × *g* for 4 hours at 4°C. Then, the pellets were laid on top of a 30% sucrose/D_2_O cushion and ultra‐centrifuged at 100 000 × g for 1 hour at 4°C. The pellets were then resuspended in 15 mL PBS and centrifuged at 100 000 × g for 1 hour at 4°C. Finally, the pellets were resuspended in 200 µL PBS.^26^ For the exosome treatment, 20 µg/mL exosomes in 100 µL PBS were added to the NP cell culture system. After 24 hours, the NP cells were collected for further study. For the in vivo treatment, 20 µg/mL exosomes in 500 µL PBS were injected into the mice through tail vein. After 48 hours, the mice were used for further study.

### 2.11 Identification and labelling of MSCs‐derived exosomes

According to previously reported,^18^ transmission electron microscope (TEM) was used to observe purified MSCs exosomes double‐layer capsule ultrastructure. The average diameter and concentration of exosome were measured by dynamic light scattering (DLS). MSCs‐derived exosomes were labelled using a PKH26 red fluorescent labeling kit (Sigma‐Aldrich) according to the manufacture's protocol. The protein markers were detected by Western blotting, as described previously herein.

### 2.12 Cell transfection

The mimic control (mimic‐NC), miR‐410 mimic, inhibitor control (inhibitor‐NC) and miR‐410 inhibitor were purchased from Synthgene. 100 nmol/L of each item was transfected into NP cells using Lipofectamine 2000 (Invitrogen) according to the manufacturer's instructions. The NP cells were harvested for further study after 48 hours. To obtain the miR‐410 OE or miR‐410 KD exosomes, MSCs were incubated with 100 nmol/L of miR‐410 mimic and miR‐410 inhibitor for 48 hours using the same reagent. The exosomes were isolated by series centrifugation, as described previously herein.

### 2.13 qPCR

Total RNA was extracted and purified using mirVana™ PARIS™ RNA and Native Protein Purification Kit (ThermoFisher) following the manufacturer's instructions. cDNA was synthesized using a TaqMan™ Advanced miRNA cDNA Synthesis Kit following the manufacturer's protocol. qRT‐PCR was then performed using TaqMan™ Fast Advanced Master Mix (Thermo Fisher). The relative expression level of the individual genes was normalized by U6 via using 2^−ΔΔCT^ cycle threshold method. The primer of miR‐410: forward: 5′‐CGCGCGAATATAACACAGATG‐3′; and reverse: 5′‐AGTGCAGGGTCCGAGGTATT‐3′; U6: forward 5′‐GCTCGCTTCGGCAGCACAT‐3′, reverse 5′‐ATGGAACGCTTCACGAAT‐3′.

### 2.14 Luciferase assay

A fragment of NLRP3 3′UTR, including the miR‐410 binding site, was predicted via TargetScan7.2 (http://www.targetscan.org/). pMIR‐WT/Mut NLRP3 3′‐UTR‐Luc reporter plasmids were purchased from Synthgene. The 293 cells were transfected with plasmids, and miR‐410 mimic as well as mimic‐NC. After 48 hours, cells were lysed, and the luciferase activities were detected by the Dual‐Glo Luciferase Assay System (Promega) following the manufacturer's protocol. The luciferase activity was calculated by renilla. The experiments were repeated at least three times independently.

### 2.15 Statistical analysis

For statistical analysis, SPSS software was used. A Student *t* test was performed to determine the significance between two groups, one‐way or two‐way ANOVA with Bonferroni post hoc tests for multiple groups. Quantitative results are represented as the means ± standard deviation (SD). *P* < .05 was considered significant.

## 3 RESULTS

### 3.1 NP cell pyroptosis was induced by IVDD

**FIGURE 1:**
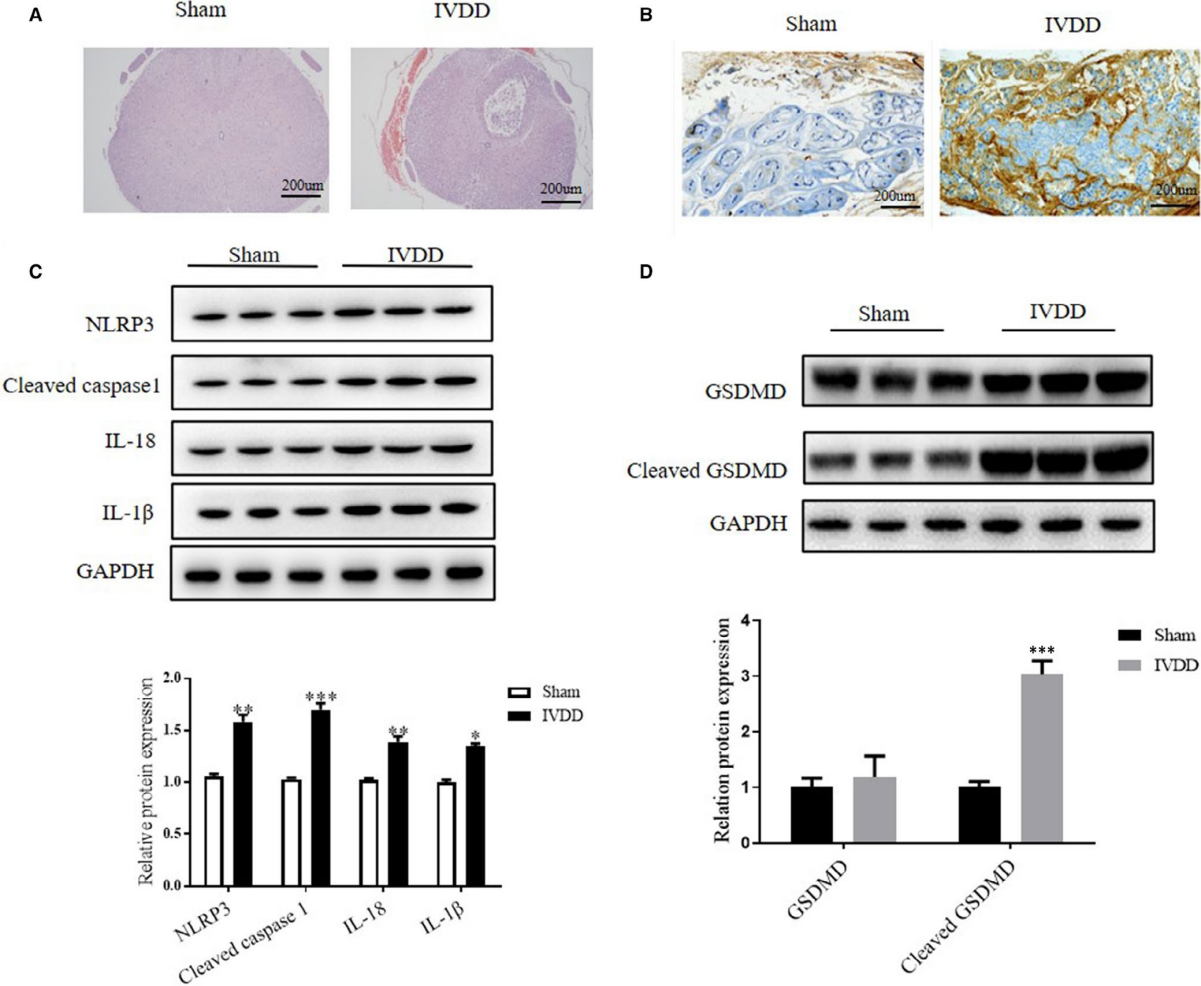
Pyroptosis occurred during intervertebral disc degeneration (IVDD). A, Representative photomicrographs of H & E stained intervertebral disc tissue of sham and IVDD mice. B, Representative photomicrographs of IHC for NLRP3 expression in sham and IVDD groups. The brown points were performed as positive signals. C and D, Representative blots and densitometric analysis were shown for NLRP3, cleaved caspase‐1, GAPDH, IL‐1β, IL‐18, GSDMD and cleaved GSDMD expression in NP tissues of three mice with sham or IVDD operation. Values are presented as mean ± SD, n = 3, *P < .05, **P < .01, ***P < .001 vs the sham group

We first determined the severity of intervertebral disc degeneration by histology assay. As shown in Figure 1A, compared with the sham group, the IVDD group showed lesser chondrocyte‐like cells and disorganized hypocellular fibrocartilaginous tissue.^27^ The expression of NLRP3 was further detected by IHC assay. As shown in Figure 1B, the NLRP3 signal in the IVDD group was significantly higher than those in the sham group. Then, we determined the protein level of NLRP3 in the NP tissues. As expected, the Western blot results showed that the level of NLRP3 was significantly up‐regulated in the IVDD group than that in the sham group. Consistently, the expression level of downstream proteins, including cleaved caspase‐1, IL‐18 and IL‐1β, was all markedly increased when the mice were subjected to IVDD (Figure 1C). Consistently, the protein level of GSDMD cleavage was remarkably increased by IVDD (Figure 1D). These data indicated that NLRP3‐mediated pyroptosis was activated during IVDD formation.

### 3.2 Effect of MSCs on pyroptosis in IVDD model

**FIGURE 2:**
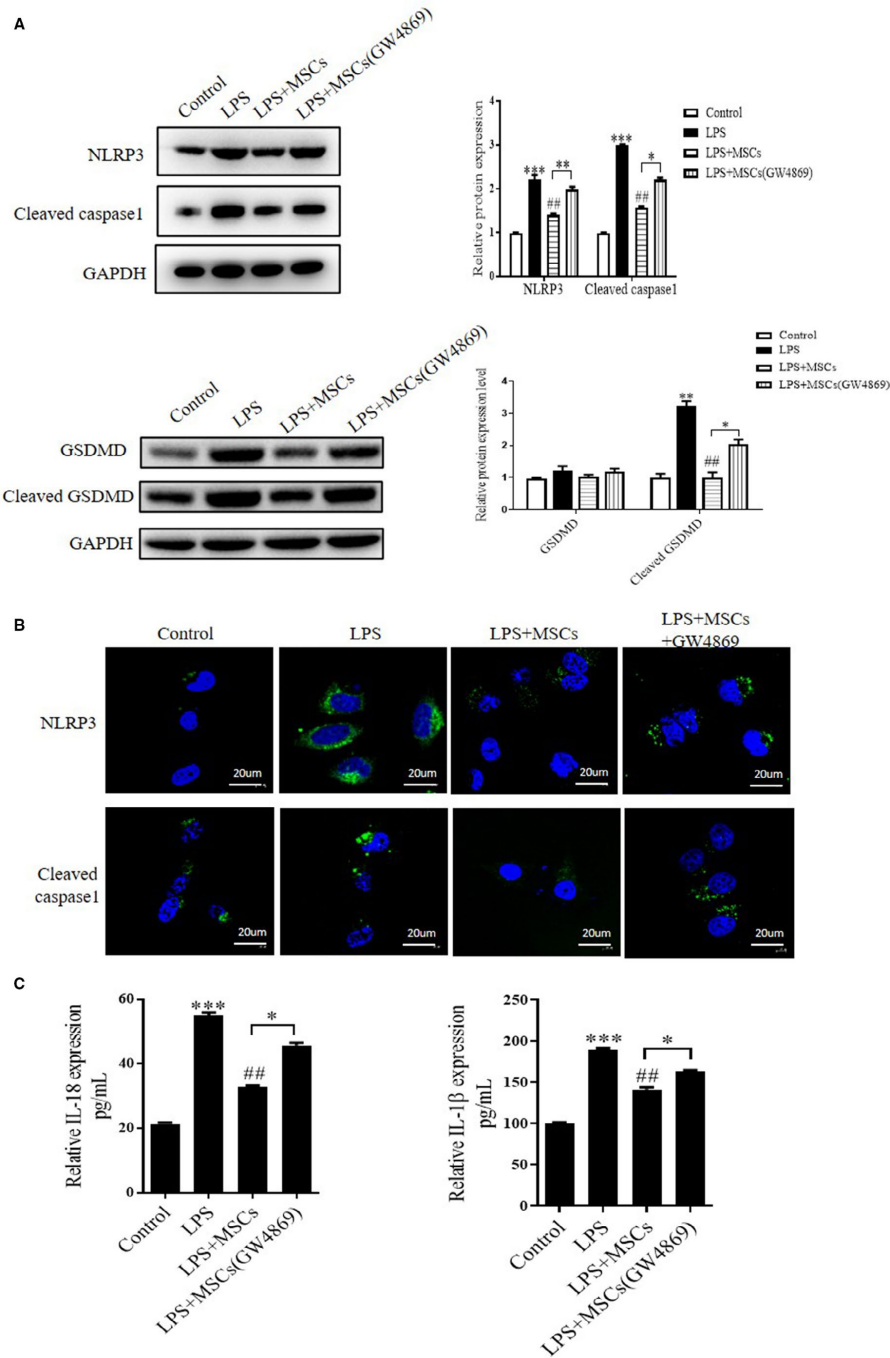
MSCs treatment inhibits NLRP3‐mediated pyroptosis in the LPS‐treated NP cells. A, Representative blots and densitometric analysis are shown for NLRP3, Cleaved caspase‐1, GSDMD and cleaved GSDMD in control, LPS, LPS + MSCs and LPS + MSCs + GW4869 groups. B, Representative photomicrographs of immunofluorescence assay for NLRP3 and Cleaved caspase 1 expression in the NP cells after different treatments. C, Quantitative analysis for IL‐1β and IL‐18 in NP cell culture supernatants under different conditions. Values are presented as mean ± SD, n = 3, *P < .05, **P < .01, ***P < .001 vs the sham group. #*P* < .05, ##*P* < .01, ###*P* < .001 vs the LPS group

To investigate the effect of MSCs on NLRP3 inflammasome activity, LPS‐treated NP cells were co‐cultured with MSCs. The result showed that LPS treatment significantly increased the expression level of NLRP3, caspase‐1 and cleaved GSDMD in NP cells. However, this phenomenon was dramatically reversed after co‐culturing with MSCs (Figure 2A). Consistently, similar results had been detected by immunofluorescence (Figure 2B). Subsequently, we evaluated the level of IL‐18 and IL‐1β expression in NP cell culture supernatants. They were increased after LPS stimulation, but both were declined when co‐cultured with MSCs (Figure 2C). It suggested that MSCs treatment inhibited LPS‐induced pyroptosis in NP cells. Interestingly, when we further treated the MSCs with GW4869 to inhibit exosomes secretion, the effect of MSCs was abolished. Moreover, this operation not only inclined the expression level of NLRP3, cleaved GSDMD and caspase‐1 but also reversed the level of IL‐18 and IL‐1β expression in LPS‐treated NP cells (Figure 2). Hence, we have been suggested that the effect of MSCs on pyroptosis might mainly cause by its derived exosomes.

### 3.3 The function of MSCs‐derived exosomes on LPS‐induced NP cell pyroptosis

**FIGURE 3:**
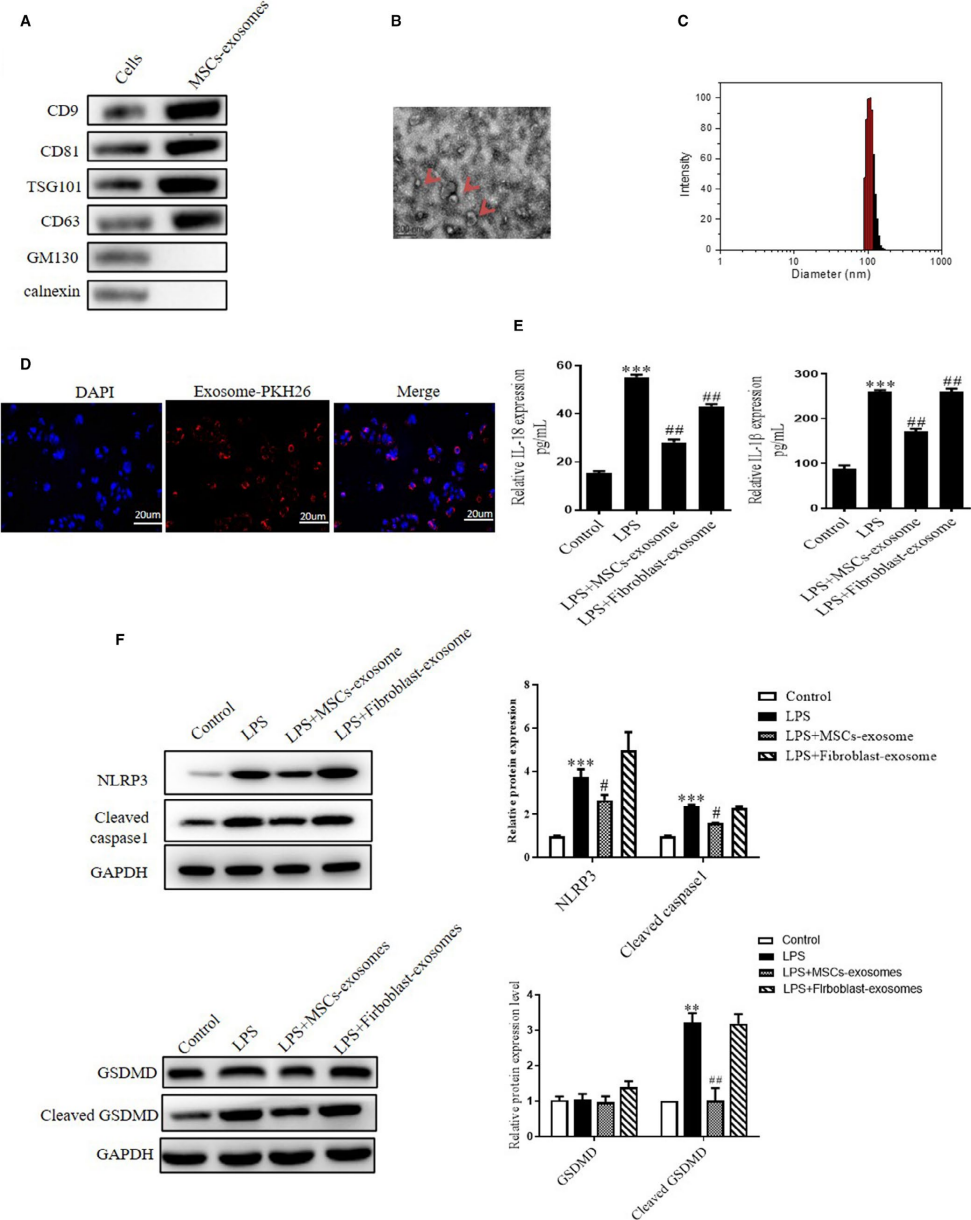
MSCs‐derived exosome treatment inhibits LPS‐induced pyroptosis in NP cells. A, Representative blot for CD63, CD9, CD81, and TSG101, GM130 and Calnexin expression in the MSCs cells and MSCs‐derived exosomes were performed by Western blot. B, The representative TEM image of exosomes. Scale bar = 200 µm. C, The size of MSCs‐derived exosomes was measured by DLS. D, Immunofluorescence indicated MSCs‐derived exosomes were taken up by NP cells. Scale bar = 20 µm. E, Quantitative analysis of IL‐1β and IL‐18 in the NP cell supernatants after different treatments were determined by ELISA. F, Western blot assay showed the expression level of NLRP3, cleaved caspase‐1, GSDMD and cleaved GSDMD in NP cells after treatment with LPS, LPS + MSCs‐exosomes and LPS + fibroblast‐exosomes. Quantification of different signal intensities was performed. Values are presented as mean ± SD, n = 3, *P < .05, **P < .01, ***P < .001 vs the control group. #*P* < .05, ##*P* < .01, ###*P* < .001 vs the LPS group

Exosomes derived from MSCs were isolated by centrifugation, and the characteristics were identified. Compared with the cell lysates, the protein levels of the positive markers such as CD63, CD9, CD81 and TSG101 were enriched in MSCs‐derived exosomes. Adversely, neither GM130 nor the Calnexin expressed in the exosomes (Figure 3A). Moreover, as shown in Figure 3B, C, the shape and size were as same as the description of previous data,^26^ with a typical size around 100 nm in diameter. Furthermore, we found the PKH26‐labelled exosomes could be uptake by NP cells after 24 hours, with a high level of red fluorescence in the cells (Figure 3D). Subsequently, we further checked the effect of exosomes on LPS‐induced pyroptosis. Compared to that in the LPS‐treated group, the LPS + MSCs‐exosome group showed a decrease in the IL‐18 and IL‐1β levels. However, the levels of these inflammatory cytokines were not changed after fibroblast‐exosome treatment (Figure 3E). Consistently, the Western blot analysis revealed that the level of NLRP3, caspase‐1 and cleaved GSDMD increased notably in NP cells after LPS treatment. However, the expression levels of these markers were significantly reduced by MSCs‐derived exosome treatment rather than fibroblast‐derived exosome treatment (Figure 3F).

### 3.4 MSCs‐derived exosomal miR‐410 inhibits pyroptosis response in NP cells

**FIGURE 4:**
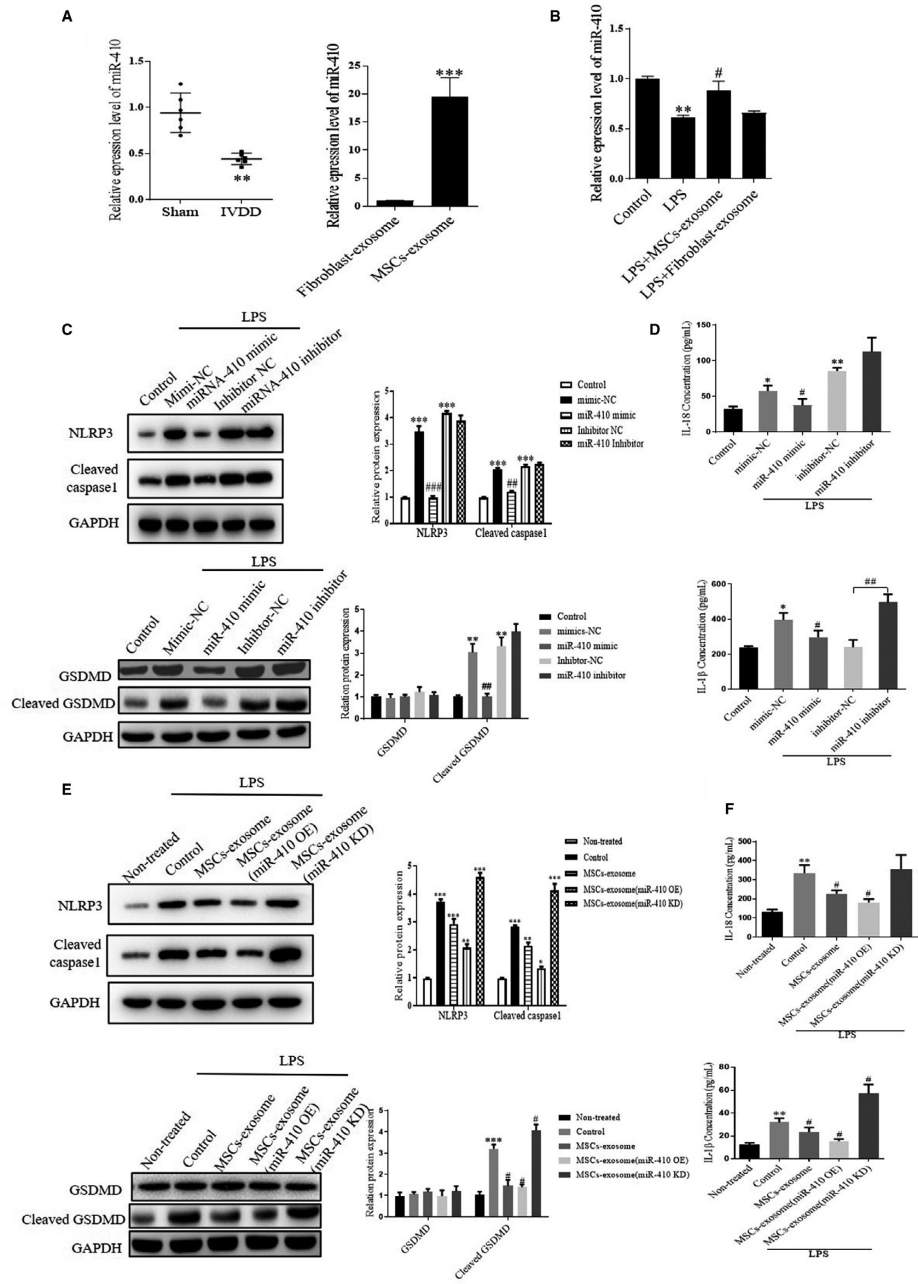
MSCs‐derived exosomal miR‐410 suppressed LPS‐induced pyroptosis in NP cells. A, The level of miR‐410 in the sham group, IVDD group, fibroblast‐exosome group and MSCs‐exosome group was analysed by qRT‐PCR. **P < .01 vs the sham group. ***P < .001 vs the fibroblast‐exosome group. B, The level of miR‐410 in NP cells with different treatment was analysed by qRT‐PCR. C, Representative blots and densitometric analysis are shown for NLRP3, cleaved caspase‐1, GSDMD and cleaved GSDMD in NP cells with different treatments. D, Quantitative analysis of IL‐1β and IL‐18 in the NP cell supernatants after different treatments were determined by ELISA. E, Cleaved caspase‐1, NLRP3, GSDMD and cleaved GSDMD protein levels in NP cells after treatment with different types of MSCs‐exosomes (with or without LPS stimulation) were detected by Western blot. F, Quantitative analysis of IL‐1β and IL‐18 in the NP cell supernatants after different treatments was determined by ELISA. Values are presented as mean ± SD, n = 3, *P < .05, **P < .01, ***P < .001 vs the non‐treated group or control group. #*P* < .05, ##*P* < .01, ###*P* < .001 vs the mimic‐NC, LPS group or inhibitor‐NC group. mimic‐NC, mimic control; inhibitor‐NC, inhibitor control; OE, mimic treatment; KD, inhibitor treatment

The expression level of miR‐410 was subsequently examined in the sham and IVDD groups. As shown in Figure 4A, significantly decreased miR‐410 level was detected in the IVDD samples as compared with the sham group. In contrast, we found that the expression of miR‐410 was dramatically higher in exosomes derived from MSCs than that in the fibroblast‐derived exosomes. Subsequently, we checked the miR‐410 level in NP cells with different treatments. As shown in Figure 4B, LPS treatment decreased the expression of miR‐410 in NP cells as compared with the control group. Moreover, comparing with fibroblast exosomes, MSCs‐derived exosome treatment re‐up‐regulated the expression of miR‐410 in LPS‐treated NP cells. Thus, our data indicated that up‐regulated miR‐410 might play a positive effect on IVDD.

To investigate the function of miR‐410, we transfected LPS‐induced NP cells with miR‐410 mimic or miR‐410 inhibitor and related controls. As expected, overexpression miR‐410 in LPS‐treated NP cells significantly inhibited pyroptosis via suppressing the NLRP3/caspase‐1 pathway. Besides, the expression level of cleaved GSDMD was dramatically reduced by miR‐410. Oppositely, transfection with miR‐410 inhibitor shown no effect on the expression level of NLRP3, caspase 1 and cleaved GSDMD (Figure 4C). Accordingly, a similar trend was exhibited in the content of IL‐1β and IL‐18 (Figure 4D). To confirm whether the exogenous miR‐410 derived from exosomes, loss or gain function experiment was performed. As shown in Figure 4E, overexpressing (OE) miR‐410 in MSCs‐derived exosomes dramatically decreased NLRP3, caspase‐1 and cleaved GSDMD protein expression in NP cells with LPS stimulation, which showed similar results with MSCs‐exosome treatment. Nevertheless, the protein levels of these markers still maintained at a high level, if the cells were treated by miR‐410 knockdown (KD)MSCs‐exosomes. The RT‐PCR analysis demonstrated that the level of IL‐18 and IL‐1β was elevated about twofolds after LPS stimulation compared with the non‐treated group, while it significantly dropped in the cells with miR‐410‐enriched exosome treatment. The silence of miR‐410 in MSCs re‐up‐regulated the expression of such cytokines in the NP cells (Figure 4F). Together, these data clearly suggested that MSCs‐derived exosomal miR‐410 might inhibit pyroptosis in NP cells.

### 3.5 Exosomal mIR‐410 is able to bind to NLRP3 directly

**FIGURE 5:**
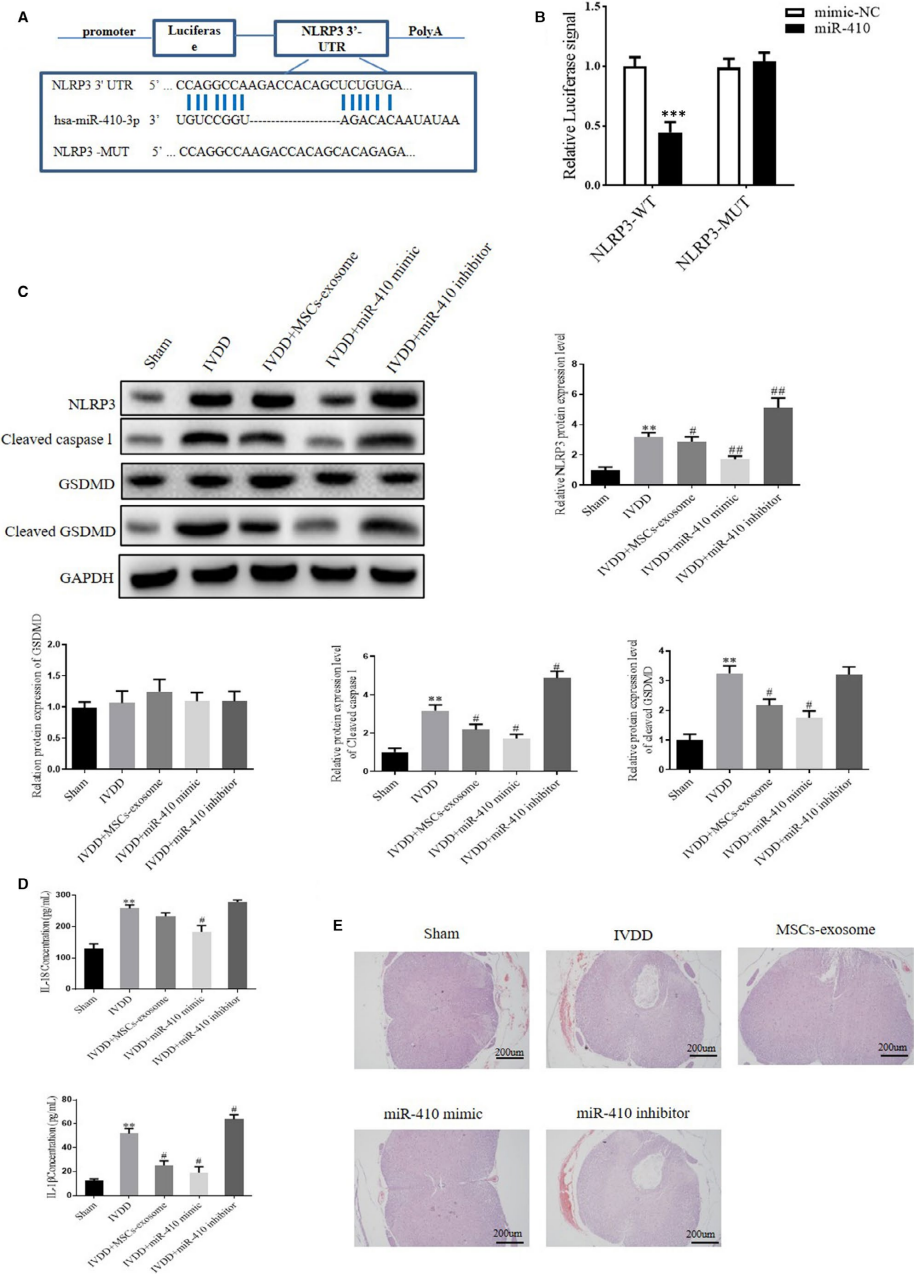
NLRP3 is a target of miR‐410. A, Sequence alignment of the NLRP3 3′‐UTR (WT and MUT) and miR‐410‐3p. B, Luciferase reporter assay was performed in 293 cells cotransfected with the plasmid containing WT or MUT NLRP3 3′‐UTRs and miR‐410 mimics or mimic‐NC. *P < .05, **P < .01, ***P < .001. C, Representative blots and densitometric analysis are shown for NLRP3, cleaved caspase 1, GSDMD and cleaved GSDMD in mimic with different treatments. D, Quantitative analysis of serum IL‐1β and IL‐18 in the mice after different treatments was determined by ELISA. E, Representative photomicrographs of H&E stained intervertebral disc tissue of mice after different treatment. Bar = 200 µm. Values are presented as mean ± SD, n = 3, *P < .05, **P < .01, ***P < .001 vs the sham group. #*P* < .05, ##*P* < .01, ###*P* < .001 vs the IVDD group. WT, wild‐type; MUT, mutant; NC, miRNA control

It is reported that multiple micro‐RNAs, including miR‐223, miR‐133 and miR‐22, could regulate NLRP3 inflammasome activation through binding to its 3′untranslated regions or post‐transcriptional regulation.^28^ To confirm the relationship between miR‐410 and NLRP3, the potential binding site of miR‐410 on the 3′UTR of NLRP3 was identified by bioinformatics analysis. The sequence of the wild‐type or mutant NLRP3 3′UTR was designed and shown in Figure 5A. miR‐410 was predicted to bind to NLRP3 3′UTR. To validate this result, a luciferase assay was performed. As shown in Figure 5B, the luciferase intensity was reduced obviously by miR‐410 mimic in the wild‐type group rather than the mutant one, which means miR‐410 is able to bind to NLRP3 directly. Next, we further discussed the role of miR‐410 in vivo. As shown in Figure 5C, MSCs‐exosome treatment significantly reversed the increased protein levels of NLRP3, cleaved GSDMD and caspase‐1 induced by IVDD. With the miR‐410 inhibitor transfection, a higher level of such markers was observed in the IVDD mice. Consistently, a similar change of IL‐18 and IL‐1β was displayed in our model (Figure 5D); in addition, MSCs‐exosomes and miR‐410 treatment alleviated the severity degree of IVDD rather than the miR‐410 inhibitor transfection (Figure 5E).

## 4 DISCUSSION

Currently, many studies have reported that NP cell death is a crucial event during IVDD formation. Two major types of cell death have been demonstrated are apoptosis and necroptosis.^29^ They were caused by the increased levels of oxidative stress or inflammatory cytokines in the degenerated IVD tissues.^30^, ^31^ The current study demonstrated whether the pyroptosis involved in the IVDD formation. We first established an IVDD model by IVD puncture and found that NLRP3‐mediated NP cell pyroptosis was activated in the progression of IVDD, with the initiation of the NLRP3 inflammasome, up‐regulation of caspase‐1 and cleaved GSDMD, as well as increased secretion of downstream cytokines IL‐18 and IL‐1β. Moreover, this study also investigated the initiation of pyroptosis as a novel participant in the LPS‐treated NP cells. With the LPS treatment, the NLRP3 was also initiated, which caused activation of caspase‐1 and GSDMD. In fact, previous studies have proved that LPS treatment could activate the inflammatory response and promote pro‐inflammatory cytokines accumulation in NP cells.^32^, ^33^ Moreover, the NLRP3‐induced pyroptosis could be activated by LPS treatment in vivo.^34^ Therefore, our data showed that NP cell pyroptosis play a vital role in the IVDD formation.

Next, the question arose on how to inhibit pyroptosis from ameliorate IVDD. Nowadays, attention has been increasingly placed on the effect of stem cell therapy. Many pieces of evidence have successfully demonstrated that MSCs have the ability of self‐renewal and differentiation into chondrocytes, osteoblasts and adipogenic lineages.^35^ MSCs based cell therapeutics showed a promised result for disc structure support, matrix synthesis in multiple animal models.^36^, ^37^, ^38^, ^39^ Our study confirmed that MSCs could inhibit NP cell pyroptosis by suppressing the expression of NLRP3 in the LPS‐induced model. However, when we further treated the MSCs with GW4869 to inhibit the secretion of exosomes, the anti‐pyroptosis effect of MSCs had been abolished. It indicated that the effect of MSCs on pyroptosis might mainly cause by its derived exosomes. Recent discovery had proved that MSCs‐derived exosomes prevented the progression of degenerative changes, although enhancing the antioxidant and anti‐inflammatory effect in NP cells or against apoptosis.^40^ However, whether they could regulate NP cell pyroptosis is unknown. Therefore, we isolated the MSCs‐derived exosomes to treat LPS‐treated NP cells. Our data showed that MSCs‐derived exosome treatment significantly decreased NLRP3 expression and reduced caspase activation, thereby suppressing the secretion of IL‐1β and IL‐18 in the NP cells under LPS stimulation. To date, accumulating evidence has explored that exosomal proteins or RNAs play a crucial role in changing the recipient's activities or functions. An essential component of exosomes, miRNAs, has been concerned increasingly in many diseases. MiR‐410 has been proven to regulate cell proliferation and apoptosis, and act as a prognostic biomarker in multiple inflammatory diseases.^41^, ^42^ Significantly increased miR‐410 expression levels have been reported to reduce the generation of cytokines, such as IL‐10, TNF‐α, IL‐1β and IL‐6.^43^, ^44^ Accumulation of oxidative stress or inflammatory cytokines in the degenerated IVD tissues was the leading cause of IVDD. It provided insight into the potential of miR‐410 in controlling IVDD formation. Moreover, compared with the healthy volunteer, miR‐410 was decreased in patients with wet age‐related macular degeneration.^45^ As another age‐related degeneration disease, IVDD represents a major clinical problem. However, the relationship between miR‐410 and IVDD remains unclear. Therefore, we focused on the role of miR‐410 in this study. In our study, a decrease in the miR‐410 level was found in our IVDD model compared with sham samples. Moreover, miR‐410 levels in MSCs‐derived exosomes were significantly higher than those in fibroblasts derived exosomes. These results provided us miR‐410 might be a potential mediator of NP cell pyroptosis. Next, we confirmed that MSCs‐derived exosomal miR‐410 significantly inhibited the pyroptosis response via suppressing the NLRP3/caspase‐1 pathway in LPS‐treated NP cells. However, this specific role of miR‐410 in pyroptosis progression we known is still limited.

NLRP3 inflammasome is a marker of pyroptosis and associated with several human chronic inflammatory diseases. NLRP3 silencing reduces a series of pyroptosis‐related markers activation.^46^ For this reason, bioinformatic analysis (www.targetscan.org) was performed to investigate the potential relationship between miR‐410 and NLRP3. It predicted that NLRP3 might be a target of miR‐410. Besides, the result of the luciferase assay confirmed this hypothesis. Thus, our results demonstrated a novel mechanism by which the down‐regulation of NLRP3 by exosomal miR‐410 administration resulted in a declined caspase‐1 and GSDMD, thereby weakening NP cell pyroptosis to alleviate IVDD. However, more evidence for the relationship between miR‐410 and NLRP3 still required. In addition, establishment of a miRNA expression profile was still essential for reveal the mechanisms of IVDD‐related NP cell pyroptosis in our future study.

In summary, we demonstrated that NP cell pyroptosis was initiated during IVDD formation. MSCs‐derived exosomes could inhibit LPS‐induced NP cell pyroptosis in vitro. In addition, MSCs‐derived exosomal miR‐410 directly binded to NLRP3 3′UTR to achieve this effect. All of our findings offer a new opportunity to prevent IVDD.

## CONFLICT OF INTEREST

The authors declare that they have no competing interests.

## AUTHOR CONTRIBUTIONS

**Jingwei Zhang:** Writing‐original draft (lead). **Jieyuan Zhang:** Visualization (equal). **Yunlong Zhang:** Conceptualization (equal); Data curation (equal); Formal analysis (equal); Funding acquisition (equal); Investigation (equal); Methodology (equal); Project administration (equal). **Wenjun Liu:** Visualization (equal). **Weifeng Ni:** Writing‐review & editing (equal). **Xiaoyan Huang:** Writing‐review & editing (equal). **Junjie Yuan:** Writing‐review & editing (equal). **Bizeng Zhao:** Investigation (equal). **Haijun Xiao:** Conceptualization (equal); Funding acquisition (equal). **Feng Xue:** Conceptualization (equal); Funding acquisition (equal).

## ETHICAL APPROVAL

The study had approval from the Ethics Committee of Southern Medical University Affiliated Fengxian Hospital.

## CONSENT FOR PUBLICATION

Not applicable.

## Data Availability

Not applicable.
